# Comparison of percutaneous vertebroplasty and conservative treatment for one level thoracolumbar osteoporotic compression fracture in a 3-year study

**DOI:** 10.1038/s41598-023-36905-8

**Published:** 2023-11-20

**Authors:** Yanchun Xie, Hongwen Gu, Yongcun Wei, Anwu Xuan, Hailong Yu

**Affiliations:** Department of Spine, General Hospital of Northern Theater Command, 83 Wenhua Rd, Shen He Qu, Shenyang, 110017 Liaoning China

**Keywords:** Health care, Health occupations

## Abstract

The efficacy of Mesh optimized versus standard percutaneous vertebroplasty (PVP) for osteoporotic vertebral compression fractures. Grid optimization (102 cases; 38 men, 64 women aged 67.3 ± 8.5) and traditional PVP groups (94 cases) were identified from 196 PVP patients treated from May 2016 to 2019. The optimal puncture site and angle forced bone cement into both groups before surgery. The main indexes were operation time, X-ray fluoroscopy times, bone cement injection volume, leakage, VAS, and injured vertebrae height. Preoperative general data were equivalent between groups (P > 0.05). All patients survived surgery without spinal cord injury, incision infection, pulmonary embolism, or death. The mesh optimization group had improved operation time (34.8 ± 6.5 min), fluoroscopy times (29.5 ± 5.5), bone cement injection volume (5.3 ± 2.1 ml), and bone cement permeability greater (3.9 percent; 4/98) than the standard PVP group (P < 0.05). Similarly, the grid optimization group had superior VAS scores (1.1 ± 0.6; 1.0 ± 0.3; and 0.9 ± 0.2) than the standard PVP group at 3 days, 3 months, and the last follow-up visit (P < 0.05). On day three after surgery, both had similar heights of injured vertebra's anterior and middle edges (P > 0.05). However, in the mesh optimization group, measurements improved to 1.8 ± 0.4 mm and (1.8 ± 0.3) mm by month three and to 1.7 ± 0.3 mm at last follow-up (P < 0.05). Mesh-optimized PVP with a mesh locator treats osteoporotic vertebral compression fractures more safely and effectively than regular PVP.

## Introduction

The prevalence rate of osteoporosis patients with the elderly over 60 years old in China has reached as high as 36% since 2016^1^. OVCF, the most frequent bone disease in middle-aged and elderly persons, can cause severe back pain, functional impairment, and mortality. Percutaneous vertebroplasty (PVP), which is safe, effective, minimally invasive, and fast-recovering, is extensively used to treat OVCF^2^.

Much research found that unilateral puncture saves surgery time, radiation exposure, soft tissue injury, and medical costs^3,4^. However, the traditional unilateral puncture technique still has the following problems: (1) Repeated "C" arm X-rays are needed to choose the skin needle entry site, spinal bone needle entry point, and needle angle. Excessive intraoperative fluoroscopy, which increases operator and patient radiation exposure; (2) Poor puncture point and angle selection will result in inadequate bone cement dispersion and postoperative discomfort; (3) Poor puncture angle damages spinal cord neuron and bone cortex, causing lower limb sensory, motor, and iatrogenic bone cement leaking^5–7^.

Accurate puncture technology reduced patient and surgeon radiation exposure and problems. All pictures used in PVP and pedicle screw placement show that the navigation system has good accuracy and minimal radiation. However, the error rate is 1.2–15.7 percent. Based on preoperative CT data, Tan et al*.*^8^ had found the entry point through planned the puncture path, as well as the best puncture angle obtained with the help of body surface angle and body surface scale. Screening and treating patients at high risk of osteoporotic fracture are cost-effective, yet around two-thirds of annual OVCFs are misdiagnosed and untreated^2^.

Based on retrospective analysis of the data of 196 patients with lumbar OVCF treated by PVP in our hospital as well as the skin being visualized by preoperative CT data, we applied a grid locator to optimize key Hemi-puncture points of PVP (skin puncture point, vertebral bony puncture point, target point of puncture needle in vertebral body) and puncture path to reduce the times of intraoperative "C" arm X-ray and find the best puncture site and angle, as well as avoiding nerve injury caused by poor puncture angle; The purpose of the comparison with traditional PVP surgery was that: (1) Discussion on the feasibility of optimizing the puncture point and puncture path of grid locator in PVP surgery before grid locator; (2) Conclusion on the advantages of using grid locator to optimize the path before operation; (3) Evaluate on the efficacy of PVP after using grid locator to optimize the path.

## Methods

### Inclusion and exclusion criteria

#### Inclusion criteria

(1) Osteoporotic and a compression fracture in the single sequence of the lumbar spine, of which kyphosis Cobb angle < 40° without neurological symptoms; (2) The patients were treated with PVP of the optimized path of grid locator before operation; (3) The comparison with the patients who received the traditional PVP operation at the same term; (4) The main observation indexes include operation time, intraoperative fluoroscopy times, pain relief degree and bone cement leakage rate; (5) The case–control study.

#### Exclusion criteria

(1) Combined spinal tumor; (2) Infectious spine diseases occur; (3) Previous history of lumbar surgery.

### General information

According to the above inclusion and exclusion criteria, 204 patients with lumbar osteoporotic compression fractures were treated with PVP from May 2016 to May 2019; a total of 196 patients were included in this study, which contains 64 males and 132 females; ages (68.3 ± 8.1 years old) with a range from 58 to 84 years old; course of diseases (2.1 ± 1.1 days) with a range from 0.5 to 5.4 days.

No Neurological symptoms occurred in the 196 patients, whose clinical symptoms were lower waist pain, tenderness, and percussion pain at the spinous process of the fracture site, Among whom 45 patients (23.0%) were unable to walk due to pain.

Wedge-shaped changes occurred in single lumbar vertebrae by X-ray and CT, as well as high signal changes by MRI T2WI. All patients had informed consent and signed the informed consent form. Moreover, all methods were carried out in accordance with relevant guidelines and regulations issued by the General Hospital of Northern Theater Command. All experimental protocols were approved by the General Hospital of Northern Theater Command approval committee.

### The selection principle of the operation method

All 196 patients were in accordance with the preoperative application of the grid locator to optimize the key puncture point and puncture path of PVP, as well as the traditional PVP operation. The advantages and disadvantages of the two methods were explained to the patients and their families in detail before the operation, which was chosen by the patients or their families.

### Case grouping

The total cases of the grid optimization group were 102, which included 38 males and 64 females, aged (67.3 ± 8.5 years old) with a range from 58 to 84 years old), the course of disease (2.3 ± 1.2 days) with the range from 0.8 to 5.4 days), among whom 73 cases had a definite history of trauma consisting of 38 cases of sprain, 33 cases of fall and 2 cases of traffic accident; the remaining 29 cases had no obvious history of trauma. Among them, 25 patients (24.5%) were unable to walk due to pain. The distribution of injured vertebrae: 59 cases of L1 vertebrae, 31 cases of L2 vertebrae, 8 cases of L3 vertebrae, 3 cases of L4 vertebrae, and 1 case of L5 vertebrae.

The traditional PVP group was in a total of 94 patients, including 26 males and 68 females, age (71.5 ± 5.6 years old) with the range from 62 to 82 years old), course of the disease (2.1 ± 1.1 days) with the range from 0.5 to 4.7 days, among whom 58 cases had a definite history of trauma, including 37 cases of sprain, 17 cases of fall, and 4 cases of traffic accident; the other 36 cases had no obvious history of trauma. Among them, 20 patients (21.3%) were unable to walk due to pain. The distribution of injured vertebrae: 52 cases of L1 vertebrae, 33 cases of L2 vertebrae, 7 cases of L3 vertebrae, 2 cases of L4 vertebrae.

### Operation method

#### Grid optimization group

**Figure 1:**
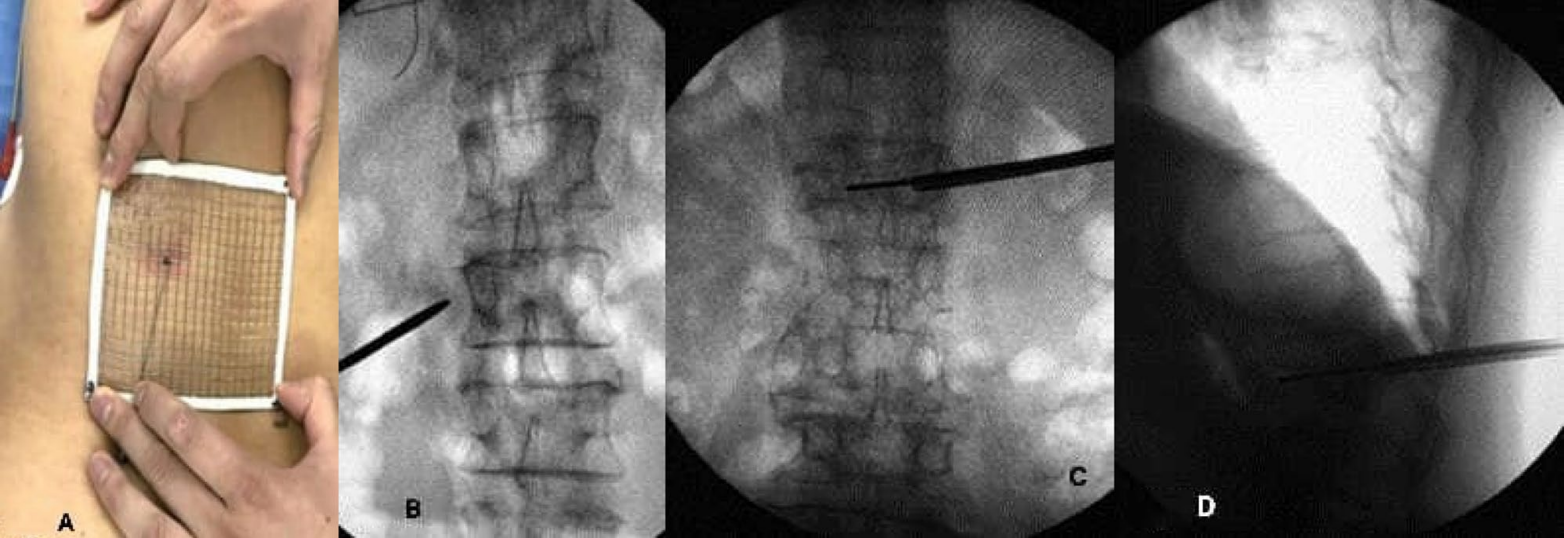
(**A**) Key puncture points of preoperative mesh optimization. (**B**) X-ray fluoroscopy showing the intersection of the midpoint of the line between the upper and lower endplates of the vertebral body and the tangent of the lateral edge of the pedicle of the vertebral body. (**C**) X-ray fluoroscopy to determine the target point in the vertebral body, where the tip of the guidewire reached the inner edge of the contralateral pedicle. (**D**) X-ray fluoroscopy showing the tip of the guidewire reaching the anterior edge of the vertebral body.

The body surface grid locator was placed on the patient's damaged vertebrae before surgery (Fig. 1A). The fractured vertebrae and body surface location were reconstructed from a CT scan of the lumbar. In addition, we were required to import 3D reconstruction data by CT into Mimics 21.0 software (Materialise company, USA), applying a new mark to label the injured vertebrae and grid locator as well as fulfilling the reconstruction of vertebrae and grid locator (Fig. 1B,C).

Cylinders replaced puncture channels after repair. The cylinder's end was placed at point C, where the extension line of the contralateral pedicle's inner border meets the vertebral body's anterior edge. After that, we had to swing the other end, which was set at point B, the midway of the line between the vertebral body's upper and lower endplates and the tangent line of the pedicle's lateral edge, which horizontally transverses it. The pen-marked skin piercing point is A (Fig. 1D).

**Figure 2:**
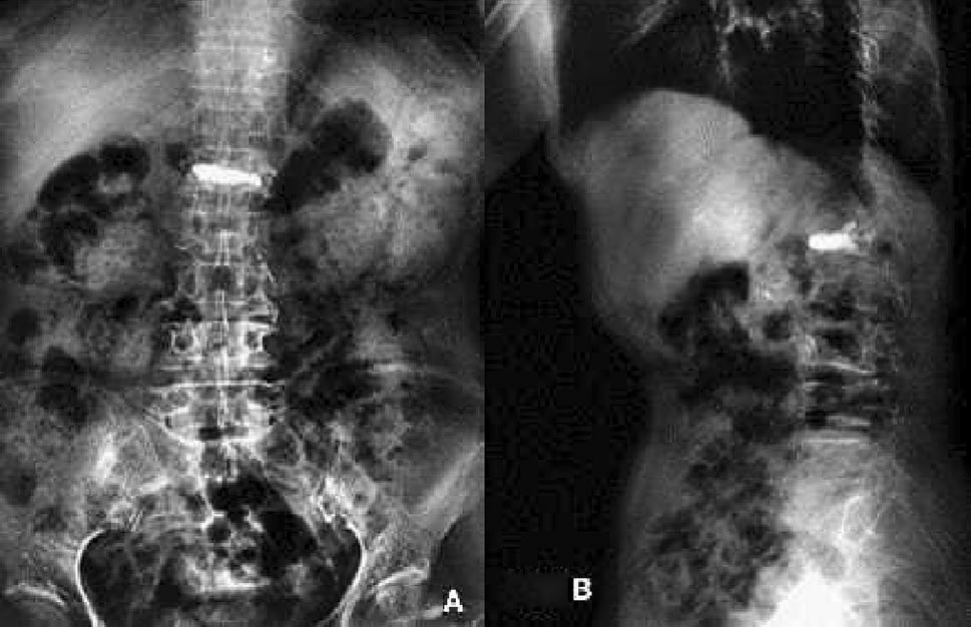
(**A**) X-ray showing that the bone cement was fully diffused into the vertebral body in the mesh optimization group, (**B**) X-ray showing the healing of injured vertebral body.

The prone patient was given local anesthesia (lidocaine with a dose of 50–100 mg per patient), sterilized, and punctured at the preoperatively planned skin puncture spot (point A) (Fig. 2A). We found the spinal bone puncture point by "C" arm X-raying the "outside edge of the pedicle" and the transverse process's tangent line (point B, Fig. 2B). The core is removed, and the guidewire is implanted when the puncture needle tip reaches the inner pedicle. The guidewire tip touches the vertebral body's anterior edge and the contralateral pedicle's inner edge at the target site (point C, Fig. 2B). After checking the puncture path and crucial puncture spots, we inserted the working channel and applied bone cement.

#### Traditional PVP group

The body position and anesthesia mode were the same as those in the grid optimization group. Under the fluoroscopy of the "C" arm X-ray machine, "9 points" (left side) or "3 points" (right side) of pedicle projection of fractured vertebral body were selected as the entry point. Anteroposterior and lateral fluoroscopy showed that the puncture needle tip did not reach the spinal canal. Thus, we continued to pierce 0.5 cm and introduced a guide wire and working channel. The lateral X-ray fluoroscopy showed the guidewire tip in the anterior and center 1/3 of the vertebral body, whereas the anteroposterior showed it in the projection of the median spinous process. Traditional PVP-targeted intravertebral guidewire tip.

#### Postoperative management

Braced patients left the bed one day following surgery. Anti-osteoporosis therapy, calcium carbonate D3, and salmon calcitonin spray were continued.

### Evaluation index

#### Operation condition

##### Operation time

To compare grid optimization group and traditional PVP group operation times, the time from disinfection of the operating area to bone cement setting was considered as min.

##### Intraoperative fluoroscopy times

The grid optimization group and typical PVP group were compared for intraoperative X-ray exposure on operators and patients by counting fluoroscopy times from the start of the procedure until puncture channel formation.

##### Amount injection of bone cement

The dose of bone cement delivered into the damaged vertebral body during surgery was measured by a scale accurate to 0.5 ml.

##### Bone cement leakage condition

Leakage has three types: type B: vertebral basilar vein to the posterior edge of the vertebral body and the anterior section of the dura mater; type S: vertebral segmental vein to the segmental vein; type C: vertebral cortical defect to any part around the vertebral body.

#### Visual analog scale (VAS)

The scale measured backache and lower limb discomfort severity. 0 represents no discomfort, and 10 is the greatest. Patients choose one of 11 numbers to indicate their discomfort, and the standard scoring is 0–10. They were scored before the operation, 3 days after, 3 months after, and at the last follow-up.

#### Height of anterior and middle edge of the injured vertebra

The damaged vertebra's upper and lower endplates were the height of its anterior edge on the lumbar lateral X-ray. After vertebral body compression fracture surgery, the injured vertebra's middle edge was the distance between the midpoints of the upper and lower endplates.

### Statistical processing

SPSS 22.0 (IBM, USA) statistical software package was used for statistical analysis. The measurement data were analyzed by the Shapior-Wilk test to determine whether it was a normal distribution. Those which were in line with normal distribution and had the same variance were expressed as (x ± s). We adopted a t-test of two independent samples as well as a chi-square test to compare the measurement data in two groups. Moreover, the t-value and p-value, along with the α value of the test level, were obtained from 0.05 on both bilateral sides.

### Ethical statement

The authors declare that the study was carried out in accordance with relevant guidelines and regulations issued by the General Hospital of Northern Theater Command.

### Study approval

All experimental protocols were approved by the General Hospital of Northern Theater Command approval committee.

## Results

### Baseline data of patients

This study first compared the preoperative baseline of osteoporotic vertebral fractures between the grid optimization group and the traditional PVP group. There were no significant differences in gender distribution, age, bone mineral density, course of the disease, height, weight, preoperative VAS score, or the height of the anterior and middle edge of injured vertebrae between the two groups (P > 0.05, Table 1). The baseline data of the two groups were comparable.

**Table 1:** Comparison of preoperative baseline data of osteoporotic vertebral compression fractures between the grid optimization group and traditional PVP group.

	Number of cases	Gender (M/F)	Age (years)	T-value of Bone marrow density (SD)	Course of disease (day)	Height (cm)	Weight (kgs)	VAS score (points)	Anterior edge of injured vertebra height (cm)	Height of middle margin of injured vertebrae (cm)
Grid optimization group	102	38/64	67.3 ± 8.5	− 4.0 ± 1.1	2.3 ± 1.2	158.2 ± 5.1	59.0 ± 3.8	7.5 ± 0.5	1.4 ± 0.4	1.4 ± 0.5
Traditional PVP group	94	26/68	71.5 ± 5.6	− 4.2 ± 0.8	1.9 ± 1.1	156.8 ± 6.8	59.3 ± 4.5	7.6 ± 0.6	1.5 ± 0.5	1.4 ± 0.7
t-value	–	0.176	0.432	− 0.769	1.135	0.248	0.147	0.683	0.592	0.558
P-value	–	0.508	0.661	0.382	0.281	0.825	0.905	0.498	0.613	0.579

### Intraoperative data

All patients completed the operation successfully. There were no serious complications such as spinal cord injury, wound infection, pulmonary embolism, or death in both group.

### Operation time

The operation time of the grid optimization group was (34.8 ± 6.5) min, which was significantly shorter than that of the traditional PVP group (42.7 ± 8.2) min (t = 1.532, P < 0.001). The operation time of the grid optimization group was 18.5% shorter than that of the traditional PVP group.

### Intraoperative fluoroscopy times

The times of intraoperative fluoroscopy in the mesh optimization group was 29.5 ± 5.5, which was better than the traditional PVP group (38.0 ± 4.0) with statistical significance (t-value = 3.251, P-value = 0.025). The times of intraoperative fluoroscopy in the mesh optimization group were lower by 22.4% than in the traditional PVP group (Table 2).

**Table 2:** Comparison of the operation time, fluoroscopy times, bone cement injection volume and bone cement permeability in patients with osteoporotic vertebral compression fractures between the mesh optimization group and traditional PVP group.

	Number of cases	Operation time (min)	Perspective time (min)	Bone cement injected volume (ml)	Bone cement permeability (%age, case)
Grid optimization group	102	34.8 ± 6.5	29.5 ± 5.5	5.3 ± 2.1	3.9 (4/102)
Traditional PVP group	94	42.7 ± 8.2	38.0 ± 4.0	4.4 ± 1.5	21.3 (20/94)
*t-*value	–	1.532	3.251	1.738	12.701
*P-*value	–	< 0.001	0.025	0.048	< 0.001

### Bone cement injection volume

**Figure 3:**
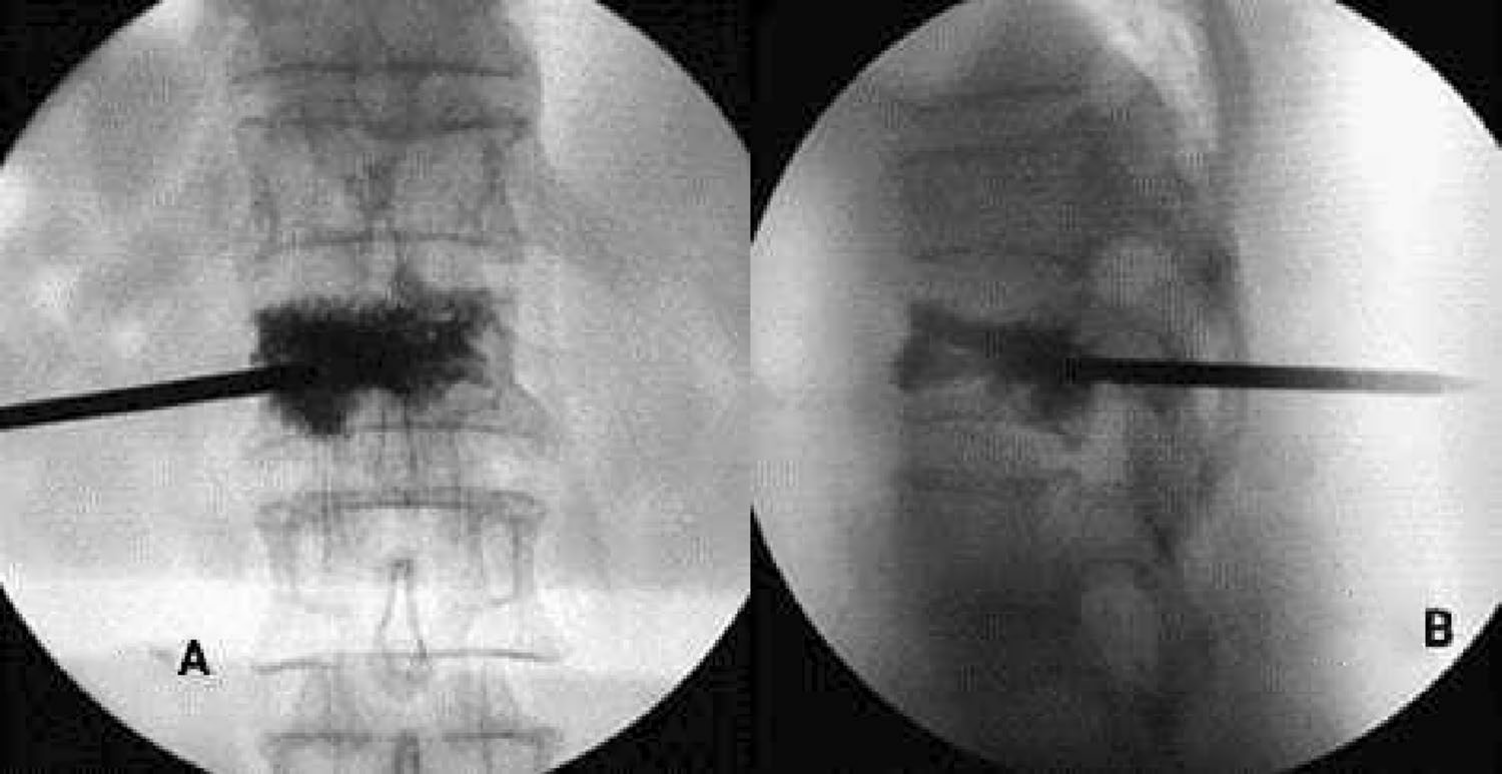
(**A**) X-ray showing that the bone cement was diffused into the puncture site in the traditional PVP group, (**B**) X-ray showing the contralateral side was not fully diffused.

In the mesh optimization group, the bone cement was fully diffused into the vertebral body (Fig. 3), of which the injection volume was (5.3 ± 2.1) ml; In the traditional PVP group, the bone cement was diffused into the puncture site, but the opposite side was not fully diffused (Fig. 3), of which the injection volume was (4.4 ± 1.5) ml. There was a significant difference in the amount of bone cement injected between the two groups (t = 1.738, P = 0.048). The amount of bone cement injected in the mesh optimization group was higher by 20.5% than in the traditional PVP group.

### Bone cement leakage rate

There were 4 cases of bone cement penetration in the mesh optimization group (3.9%) and 20 cases in the traditional PVP group (21.9%). There was a significant difference in the incidence of bone cement penetration between the two groups (χ^2^ = 12.701, P < 0.001). The penetration incidence of bone cement in the traditional PVP group was about 5.5 times higher than in the mesh optimization group.

### VAS score of low back pain

The VAS score of the grid optimization group improved to (1.1 ± 0.6) points on the 3rd day after the operation, which was (0.9 ± 0.2) points at the last follow-up more than 1 year after the operation, while the VAS score of the traditional PVP group improved to (1.4 ± 0.6) points on the 3rd day after the operation, which was (1.1 ± 0.3) points at the last follow-up more than 1 year after operation (Table 3). There were statistically significant differences between the two groups at 3 days, 3 months, and the last follow-up (P < 0.05, Table 3).

**Table 3:** Comparison of postoperative VAS score, the height of the anterior and middle edge of injured vertebra in patients with osteoporotic vertebral compression fractures between mesh optimization group and traditional PVP group.

	Number of cases	VAS score (points, cm)			Anterior edge of injured vertebrae height (cm)			Middle edge of injured vertebrae height (cm)		
Time period	–	3 days (PO)	3 months (PO)	Last follow-up (PO)	3 days (PO)	3 months (PO)	Last follow-up (PO)	3 days (PO)	3 months (PO)	Last follow-up (PO)
Grid optimization group	102	1.1 ± 0.6	1.0 ± 0.3	0.9 ± 0.2	1.9 ± 0.3	1.8 ± 0.4	1.7 ± 0.3	1.9 ± 0.6	1.8 ± 0.3	1.7 ± 0.3
Traditional PVP group	94	1.4 ± 0.6	1.3 ± 0.5	1.1 ± 0.3	1.8 ± 0.4	1.6 ± 0.3	1.5 ± 0.4	1.8 ± 0.2	1.7 ± 0.5	1.5 ± 0.3
*t-*value	-	2.082	1.624	4.326	0.731	2.514	2.172	0.601	0.931	1.983
*P-value*	-	0.005	< 0.001	< 0.001	0.438	0.018	0.021	0.572	0.039	0.004

*PO* Post Operative.

### The height of the anterior and middle edge of the injured vertebrae (cm)

In the mesh optimization group, the height of the anterior edge of the injured vertebra recovered to (1.9 ± 0.3) cm on the 3rd day after the operation, which was maintained at (1.7 ± 0.3) cm at the last follow-up, more than 1 year after the operation; In the traditional PVP group, the height recovered to (1.8 ± 0.4) cm on the 3rd day after the operation, which maintained at (1.5 ± 0.4) cm at the last follow-up more than 1 year after the operation. In addition to the third day after the operation, the height of the anterior edge of the injured vertebra in the mesh optimization group was superior to the traditional group at 3 months after the operation, and the last follow-up with statistically significant. (P < 0.05, Table 3).

In the mesh optimization group, the height of the middle edge of the injured vertebra recovered to (1.9 ± 0.6) cm on the third day after the operation, which remained at (1.7 ± 0.3) cm at the last follow-up more than one year after the operation; while the traditional PVP group recovered to (1.8 ± 0.2) cm on the 3rd day after the operation, which maintained at (1.5 ± 0.3) cm at the last follow-up more than 1 year after the operation. In addition to the third day after the operation, the height of the middle edge of the injured vertebrae in the mesh optimization group was higher than the traditional PVP group 3 months after the operation and the last follow-up with statistically significant (P < 0.05, Table 3).

## Discussion

Fluoroscopy is not required for skin entry point selection. The grid recognizes your point on the skin surface as the intersection of horizontal and vertical lines. After choosing point A, experienced doctors can puncture fast. Mesh optimization had a considerably lower fluoroscopy frequency than a puncture.

PVP was used to treat 196 OVCF patients, and mesh optimization reduced fluoroscopy and operation time. The frequency and time of single puncture fluoroscopy in vertebroplasty are mainly used to select the skin entry point (point A) and bone entry point (point B). The traditional single puncture approach involves an expert operator selecting the skin entrance point under fluoroscopy and the bone entry point by altering the needle angle. However, if the puncture needle angle is not correct to enter the vertebral body through the pedicle, we must reselect the skin entry site and vertebral bone entry point, increasing fluoroscopy frequency and worsening soft tissue and joint capsule injury. According to the rebuilt preoperative CT data of the fractured vertebral body, we designed the puncture needle target point (point C) and bone entrance point (point B) to limit unilateral puncture fluoroscopy and save operation time^9^. We also created a reverse extension line to the skin surface through the connection between points C and B to find the skin entry location (point A), which a preoperative body surface locator may validate without fluoroscopy. In the selection of vertebral bony puncture point (point B), Yan et al.^10^ proposed that In unilateral puncture, the puncture point should be 5 mm outside the pedicle and the transverse process midpoint, while Li Yuwei et al.^11^ proposed that Puncture the transverse process tip about 10 mm outside the pedicle. This study concludes that 5 mm or 10 mm on the outer edge of the pedicle could not be determined by intraoperative fluoroscopy. According to the previous study^12^, the width of the base of the segmental pedicle and vertebral body in thoracic 4-lumbar 5 gradually increased. In this investigation, we picked the middle tangent of the transverse process and the "outside edge of the pedicle" after moving one distance horizontally as the vertebral body's bone puncture site (point B). The vertebral bone needle entry location reduces fluoroscopy frequency and operation time because the anatomic reference under fluoroscopy is clearer during puncture.

The mesh optimization group had significantly lower VAS scores at 3 days, 3 months, and 1 year after surgery than the standard puncture group; however, the height of the anterior and middle vertebral bodies was much higher.

Bone cement poured into the conventional vertebral body model is distributed spherically from the center to the surrounding area^13^. Xie et al.^14,15^ reported that the vertebral body midline had greater clinical efficacy following vertebroplasty due to bone cement dispersion. In order to fully disperse the bone cement to the contralateral side, we chose the intersection of the inner border of the contralateral pedicle and the lateral front of the vertebral body as the puncture path's target point (point C). According to the biomechanical study of bone cement by Chevalier et al.^10^, it was suggested that When bone cement contacted both upper and lower endplates, vertebral body stiffness increased by 8 times and strength by 11 times, while when it only contacted one endplate, stiffness increased by 2 times and strength did not rise appreciably. Point C was equidistant between the upper and lower endplates, making bone cement diffusion easier and strengthening the broken vertebral body. Thus, the grid optimization group outperforms the puncture group in postoperative discomfort, vertebral body height, and bone cement injection.

Unilateral puncture needles should target the anterior and center 1/3 of the vertebral body, according to previous studies. There are also studies^16^ that reported that the dense venous plexus distribution in the anterior and middle 1/3 of the vertebral body was injected with bone cement to promote venous leakage^17^. The collapse of the endplate above OVCF caused 42% of bone cement leakage, according to a previous study. Thus, we targeted the contralateral pedicle medial edge and vertebral body lateral front cortical intersection (point C). This avoids the dense vertebral venous plexus distribution and makes the puncture path equal to the vertebral body's upper and lower endplates, reducing bone cement leaking from the top endplate. This study's bone cement leakage rate was 3.9 percent, compared to the typical group's 21.3 percent. Compared with the traditional PVP operation, the mesh-optimized PVP operation with a mesh locator is safer and more effective in the treatment of osteoporotic vertebral compression fractures.

## Data Availability

The data used to support this study are available from the corresponding author upon request.
